# Patient safety culture measurement in general practice. Clinimetric properties of 'SCOPE'

**DOI:** 10.1186/1471-2296-12-117

**Published:** 2011-11-01

**Authors:** Dorien LM Zwart, Maaike Langelaan, Rosalinde C van de Vooren, Marijke M Kuyvenhoven, Cor J Kalkman, Theo JM Verheij, Cordula Wagner

**Affiliations:** 1 Department of General Practice, Julius Center for Health Sciences and Primary Care, University Medical Center Utrecht, The Netherlands; 2 Nivel, Netherlands Institute for Health Services Research, Utrecht, The Netherlands; 3 Patient Safety Center, University Medical Center Utrecht, The Netherlands; 4 EMGO+ Institute, Department of Public and Occupational Health, VU University Medical Center, Amsterdam, The Netherlands

## Abstract

**Background:**

A supportive patient safety culture is considered to be an essential condition for improving patient safety. Assessing the current safety culture in general practice may be a first step to target improvements. To that end, we studied internal consistency and construct validity of a safety culture questionnaire for general practice (SCOPE) which was derived from a comparable questionnaire for hospitals (Dutch-HSOPS).

**Methods:**

The survey was conducted among caregivers of Dutch general practice as part of an ongoing quality accreditation process using a 46 item questionnaire. We conducted factor analyses and studied validity by calculating correlations between the subscales and testing the hypothesis that respondents' *patient safety grade *of their practices correlated with their scores on the questionnaire.

**Results:**

Of 72 practices 294 respondents completed the questionnaire. Eight factors were identified concerning *handover and teamwork, support and fellowship, communication openness, feedback and learning from error, intention to report events, adequate procedures and staffing, overall perceptions of patient safety *and *expectations and actions of managers*. Cronbach's alpha of the factors rated between 0.64 and 0.85. The subscales intercorrelated moderately, except for the factor about intention to report events. Respondents who graded patient safety highly scored significantly higher on the questionnaire than those who did not.

**Conclusions:**

The SCOPE questionnaire seems an appropriate instrument to assess patient safety culture in general practice. The clinimetric properties of the SCOPE are promising, but future research should confirm the factor structure and construct of the SCOPE and delineate its responsiveness to changes in safety culture over time.

## Background

Patient safety has rapidly become an important issue in primary health care. Numerous studies have revealed that harmful incidents do occur in general practice [1-9]and it is generally estimated that around 50% of adverse events in health care can be prevented [10-12]. A major challenge in addressing the safety issues in general practice is to create a culture for safety which has not been established yet [13].

A constructive patient safety culture is recognised as a key condition for improving patient safety [14,15]. The safety culture of a healthcare organisation is defined as the product of individual and group values, attitudes, competencies, and patterns of behaviour. These, in turn, influence an organisation's commitment to safety, as well as the style and proficiency of safety management [16]. There is an ongoing debate on the validity of quantitatively measuring culture; surveys may only assess 'climate' which refers to individual opinions and attitudes, rather than 'culture' which concerns underlying shared values and assumptions [17,18]. We acknowledge the value of this distinction between 'climate' and 'culture'. However, as both terms are used interchangeably in different publications and this discussion does not immediately affect our results, we only applied the term 'culture' in this paper.

Organisations with a positive safety culture are characterised by communication founded on mutual trust and openness, by shared perceptions of the importance of safety, and by organisational learning and confidence in the efficacy of preventative measures [16,19]. Indeed, in other industries a positive safety culture was associated with both higher employee safety compliance [20] and a better organizational performance [21] and recent publications suggest similar relationships in healthcare [22,23]. An additional reason for aspiring to a positive safety culture is that it could stimulate incident reporting and analysis by professionals which is a fruitful tool for safety improvement [24,25].

To establish a positive safety culture in general practice, a first step is to evaluate the current patient safety culture. As at the time of the start of this study (2006) all available surveys originated from hospital care [26-28], the aim of this study was to adapt the Dutch translation of the Hospital Survey on Patient Safety Culture (HSOPS) of the Agency for Healthcare Research and Quality (AHRQ) for use in Dutch general practice, and to investigate the internal consistency and construct validity. The adapted questionnaire was called 'SCOPE' which is an acronym in Dutch for systematic culture inquiry on patient safety in primary care.

## Methods

### Developing the questionnaire

The questions included in the SCOPE were derived from the Dutch translation of the HSOPS [28]. The Dutch HSOPS measures 11 dimensions of patient safety culture: Teamwork across hospital units, Teamwork within units, Adequate shift changes, Frequency of event reporting, Non-punitive response to error, Communication openness, Feedback and communication about error, Supervisor/manager expectations and actions promoting patient safety, Hospital management support for patient safety, Adequate staffing and Overall perceptions of safety.

In order to develop the SCOPE, questions from the Dutch HSOPS were adjusted for the primary care setting if necessary. The adjustments were made in an iterative process of independent assessments, firstly between authors, secondly among a pre-test panel of 5 general practitioners (GPs), 2 GP-trainees, 3 administrative-medical nurses (MNs) and 1 expert in psychometric methods, and finally again among authors. The panel did not meet in person, but communicated by e mail, or individually with a researcher (RvdV). Examples of adjustments: the word *hospital unit *was changed into *GP practice*. Three items from Dutch HSOPS were deleted based on discussion about face validity with the expert-professionals from general practice ('Patient safety is never sacrificed to get more work done', 'There is good cooperation between hospital units that need to work together' and ' It is often unpleasant to work with staff from other hospital units'), and seven new items were added to SCOPE (marked in the table in the additional file 1). After final consensus on the adaptations, the research version of the SCOPE consisted of 46 questions about patient safety culture. All patient safety items used five-point Likert response scales of agreement (strongly disagree 1 to strongly agree 5) or frequency (never 1 to always 5). In addition, two questions were formulated regarding the frequency of adverse event reports in the last twelve months and a patient safety grade. The wording of the latter question was "Please, give your practice a general assessment for patient safety". The response options included: 1: failing, 2: poor, 3: acceptable, 4: good and 5: excellent. Finally, a number of items assessed work-related information, e.g. the respondent's function, experience and how many hours/week the respondent worked within this practice.

### Data collection and participants

The SCOPE was implemented in the web system for GP practice accreditation of the Dutch College of GPs [29]. This web system could be accessed by the 470 Dutch GP practices which were currently being assessed for quality to acquire accreditation. The practices were requested to fill out the SCOPE questionnaire on a voluntary basis, as part of their ongoing accreditation process. It was not to be expected that all potential practices would participate, as SCOPE was not obliged for acquiring accreditation. At the time of the survey, most GP practices did not have a reporting procedure; SCOPE was offered as a voluntary first step towards concretizing safety management requirements.

Since February 15, 2009 the SCOPE questionnaire could be optionally approached by the 3268 caregivers of these practices. In the Dutch primary care setting GPs, medical assistants and practice nurses work closely together in small practice teams. Besides diagnosing and estimating the urgency of new health problems, GP practices offer preventive services, geriatric and paediatric support, management of chronic diseases and minor surgery. The GPs provide the medical care and usually are responsible for practice management. The medical assistants and practice nurses execute both administrative or organisational tasks and preventive medical care. In some practices physiotherapists and managers are part of the practice teams as well. Because SCOPE aimed at assessing safety culture in general practice, the practice teams were chosen as study population. Hence, in each practice all staff, including GPs, MNs, practice managers and physiotherapists were asked to participate.

The data were automatically stored in a database. After a pre-agreed period of five months the dataset for this study was converted into a format that can be read by the Stata software [30] and all possibly identifying features were removed in order to assure anonymity for the participants. Because this study did not involve patients, formal ethical approval was not needed for this study according to Dutch law.

### Initial analyses

Respondents with more than 50% missing values were excluded from analysis. When a respondent chose two or more options at one item, this item was marked as missing. Distribution properties of responses to the items for the SCOPE were studied by examining missing values, ceiling and floor effects. Items with more than 50% missing responses were deleted, because they could not add sufficient information. We chose this liberal percentage of 50% because we did not want to delete potentially relevant items. We deleted items with ceiling or floor effects > 75%, because they do not contribute sufficiently to the assessment of patient safety culture. Missing values of remaining items were imputed by a hot-deck procedure: the average value of a subset of comparable cases (respondents with the same profession, same working hours, and being part of management or not) was imputed for the missing values. Since the questionnaire contained positively as well as negatively worded items, the latter were recoded to make a higher score always a more positive response.

## Statistical analyses

### Factor analysis

Factor analysis was used to explore the different underlying factors (i.e. subscales). These subscales may represent aspects for the development of a positive patient safety culture. As the factor structure of the Dutch HSOPS could not be confirmed because of deleted and rephrased items, we performed an explorative factor analysis (principal component analyses with promax-rotation) in Stata to decide on the number of factors. Two steps were completed to decide on the number of factors: [1] the eigenvalue is larger than one, and [2] inspection of the scree plot. If the eigenvalue for a factor is larger than one, it explains at least as much variance as a single variable. The disadvantage of limiting to eigenvalues is that it often results in too many factors. Therefore, we used the scree plot as a supplementary method. The number of factors is defined by the cut-off point at which the slope of the scree plot approaches zero [31]. Items with a factor loading less then 0.40 on all factors were excluded. Furthermore, items that loaded on more than one factor were excluded. Each factor should be comprised of at least three items [32]

### Internal consistency

The reliability of each subscale is investigated by calculating measures which indicate the internal consistency (i.e. homogeneity) of the items that form the subscale. It is assumed that questions belonging to the same underlying domain will correlate as they measure the same aspect of patient safety culture. First, the internal consistencies of the subscales were examined using Cronbach's α, a value between 0 and 1. An alpha larger than 0.6 was considered acceptable; this indicated that different items supposed to measure the same concept [33]. In contrast to alpha, the average inter-item correlation is independent of the number of items and sample size when measuring internal consistency. Therefore, we also checked whether the inter-item correlations, aiming at an average inter-item correlation ranged between 0.20-0.70. Finally, item-rest correlations between individual items and the sum of the remaining items on a factor were calculated. Items with an item-rest correlation of 0.20 or less were excluded [34].

### Construct validity

Construct validity deals with the question of whether the empirical findings correspond with theoretical expectations concerning the questionnaire. Hypotheses about relations with other measures or variables should be postulated [35,36]. Our first postulated hypothesis was that respondents who graded patient safety in their practice highly also scored significantly higher on the subscales of the SCOPE than respondents grading patient safety lower. A second hypothesis was that respondents who answered that they reported one or more incident in the past year have significantly higher scores on the factor *"communication openness about incidents" *than respondents who never reported an incident. Furthermore, construct validity would be supported if the subscales intercorrelate moderately. High correlation between subscales (r > 0.70) would indicate that these subscales measure the same underlying aspect of safety culture. Therefore, Pearson correlation coefficients between the subscales were calculated.

## Results

A total of 331 respondents from 72 practices with estimated 497eligible participants (response rate 67%) returned the questionnaire between February and July 2009. Of the 331 respondents 294 (88.8%) completed the questionnaire. Thirty-seven did not fill out at least 50%. They were all excluded from further analyses. Fifty-eight percent of the respondents had no missing values. There were no questions with more than 50% missing values.

Seventy-three of the 294 respondents worked as a general practitioner (25%). The remaining respondents worked as medical-administrative assistant (60%) or practice nurse (15%). The GPs were representative concerning age and work experience for the general Dutch GP population. Female GPs, part timers and group practices were overrepresented among our respondents. (table 1[37]).

**Table 1:** Characteristics of participating medical assistants and practice nurses (MNs), GPs and practices compared to all Dutch GPs and practices

Characteristics of participants		Study population (n = 294)		National demographic data GPs
		
		MNs * (n = 221)	GPs (n = 73)	GPs **(n = 8766) **[37]
**Gender**	male	1.2	51.4	62.2
	
	female	98.8	48.6	37.8

**Age (years)**	< 39	47.2	19.3	20.9
	
	40-49	32.6	27.4	30.7
	
	50 and older	20.4	53.4	48.3

**Experience** **(years of practice)**	0- 10	76.9	47.9	60.2
	
	11-20	16.7	27.0	21.3
	
	21-30	5.2	22.5	15.8
	
	More than 30	0.8	3.0	2.8

**Working hours (fte)**	0-0.4	21.9	9.0	9.4
	
	0.5-0.6	43.9	20.9	20.9
	
	0.7-0.8	20.8	37.4	15.2
	
	0.9-1.0	13.2	33.0	54.5

**Practice organisation**	solo	19.9	12.4	18.0
	
	duo	23.9	23.7	28.2
	
	group practice or health care centre	56.1	63.9	53.8

(Reference data about medical assistants and practice nurses (MNs) are not available; * the figures shown are the distributions found in all MNs that responded to SCOPE in web system (n = 1100). The specific figures of the 221 MNs of this substudy could not be retraced.)

### Explorative factor analysis

After initial factor analysis in Stata, examination of eigenvalues and the scree plot indicated eight factors (data not shown). Following factor extraction and promax rotation, the eight factors accumulatively accounted for 53% of the total variance.

The table in the additional file 1 gives the mean scores with standard deviations and factor loadings per item. Three items were deleted because the factor loadings were too low; one item of the original Dutch HSOPS questionnaire was eliminated ('After we make changes to improve patient safety, we evaluate their effectiveness') and also two newly added items ('Important patient care information is often lost during shift changes from day-care to out-of-hours care ' and 'Important patient care information is often lost during shift changes from out-of-hours care to day-care '). This resulted in a questionnaire with 43 items, grouped in eight dimensions.

The internal consistency, calculated for every factor, was acceptable 0.64 < alpha < 0.85. The average inter-item correlation was 0.37 and item-rest correlations ranged from 0.28 to 0.78.

### Construct validity

All subscales, except for factor 5, correlated moderately with each other. The highest correlations were found between factor 2 *Support and fellowship *and factor 3 *Communication openness *(r = 0.57) and between factor 3 *Communication openness *and 6 *Adequate procedures and adequate staffing *(r = 0.60). Factor 5 '*Intention to report events *' correlated weakly with factor 4 '*Feedback about and learning from error' *(r = 0.24) but did not correlate at all with the remaining subscales. (Table 2).

**Table 2:** Mean subscale scores, correlation with *patient safety grade *and intercorrelations between the eight subscales (Pearson correlation coefficient r)

Dimension	Mean	SD	Correlation with patient safety grade	1	2	3	4	5	6	7
1 Handover and teamwork	3.84	0.48	0.28							

2 Support and fellowship	3.93	0.47	0.23	0.28						

3 Communication openness	3.97	0.50	0.32	0.30	0.57					

4 Feedback about en learning from error	4.03	0.61	0.34	0.23	0.36	0.38				

5 Intention to report events	3.87	0.88	0.12	0.14	0.03	0.04	0.24			

6 Adequate procedures and adequate staffing	3.83	0.49	0.41	0.39	0.41	0.60	0.29	0.01		

7 Overall perceptions of safety	3.66	0.51	0.34	0.30	0.40	0.32	0.36	0.04	0.40	

8 Supervisor/manager expectations/actions	3.81	0.52	0.26	0.30	0.49	0.51	0.30	0.05	0.44	0.52

According to our postulated hypothesis, respondents with higher *patient safety grades *scored higher than the other respondents for seven out of eight factors of the SCOPE. The highest correlation of experienced patient safety grade was with factor 6 *Adequate procedures and adequate staffing *(r = 0.41; p < 0.001). Factor 5 *Intention to report events *did not correlate with *patient safety grade *(r = 0.12; p < 0.05).

No correlations were calculated with the item *number of events reported*, because of the lack of variability and skewed nature of this item (90% of the respondents indicated not to have reported any incidents during the last twelve months).

## Discussion

In the eight-factor model, the internal consistency of the factors and the construct validity of the SCOPE questionnaire were mostly satisfactory. The construct validity was sufficient for all subscales, except for the subscale regarding intention to report incidents which correlated poorly with other subscales. The hypothesis that the patient safety grade that respondents gave for their practice would correlate positively with their scores on all factors, was confirmed, except for factor 5. The hypothesis about a positive correlation between incident reporting and communication openness could not be tested because most respondents never had reported an incident before.

This study has several limitations. Participants were not randomly selected from the study population. Instead, the set of data collected in the first months of the data collection was used for studying the clinimetric properties of the questionnaire. Also, among respondents there were more women and slightly more part time working GPs in group practices than in the average Dutch GP practice. This probably follows from the study population being involved in practice accreditation. In addition, it is likely that participating practices, both involved in quality assessment and willing to evaluate their safety culture, may have a more open culture than the average Dutch GP practice. Hence, the results on the questionnaire might be biased by selection. However, the influence of a possible selection bias on the study results would not be important because, in theory, the construct of patient safety culture in general practice does not change in different study populations. Nonetheless, both cross validation of factor solutions and confirmative factor analysis is recommended to confirm the factor structure that we found.

The eight factor structure partly corroborates the original eleven-factor-structure of the Dutch-HSOPS. The domains of factors of the SCOPE were comparable to those of the Dutch HSOPS. However, SCOPE lacked a factor concerning '*teamwork across units*', and combined most items about teamwork and handover in one factor. Also, the factors *communication openness *and *non-punitive response to error *of Dutch -HSOPS combined in SCOPE. In addition, the items about management, staffing and overall perception of patient safety which constituted four factors in the Dutch HSOPS, were structured in three factors in SCOPE. Only factor 5 *Intention to report events *in SCOPE was exactly the same as in the Dutch-HSOPS.

The revised factor structure seems more readily interpretable for use in general practice. Most differences can be explained by the differences in organisation and the smaller scale of GP practices, compared to hospitals. In contrast with Dutch-HSOPS, factor 5 *Intention to report event' *only correlated with one other factor in the SCOPE. This may indicate that, as incident reporting in Dutch general practice is still very uncommon, it is currently not perceived as an integral part of a patient safety culture in GP practices. Yet, we decided to maintain the subscale about intention to report because it is likely that some form of incident reporting will increase in general practice as part of future patient safety interventions and will become part of a patient safety culture in general practice.

We acknowledge that revising the HSOPS into SCOPE hampers comparison between secondary and primary care, because most factors consist of different items. However, one also could conclude from the different factor structures that safety culture in both settings contains slightly different concepts. This would make comparison irrelevant. The contrast between the generally favourable responses on the subscales and the very low self-reported numbers of incident reports is striking. It probably reflects the general absence of incident reporting systems Dutch general practice at the time we collected our data [38,39]. At the time of our study formal safety management was in its infancy in Dutch general practice. However, it rapidly developed in the last two years and follow up research concerning changes in patient safety culture would be highly relevant.

However, whether SCOPE has the ability to capture changes in patient safety culture resulting from interventions aimed at improving patient safety within general practice has not been determined yet. The appropriateness of SCOPE to measure such changes should be part of further research on its responsiveness to change [36]. This should also include statistical analysis at the practice level, because it is likely that safety culture is influenced within practices.

## Conclusions

In conclusion, we have revised the Dutch-HSOPS for use in general practice. The SCOPE seems an appropriate instrument to assess patient safety culture in general practice. The clinimetric properties of the SCOPE are promising, but future research should confirm the factor structure of the SCOPE and scrutinise its validity and responsiveness to change.

## Competing interests

The authors declare that they have no competing interests.

## Authors' contributions

DLMZ initiated the research project, was involved in the collection, the analyses and interpretation of the data, and wrote the manuscript. ML performed the statistical analyses of the data and co-wrote the manuscript. RCV was involved in the collection and interpretation of the data. MK contributed to the analysis and has been involved in revising the manuscript. CJK, TJMV and CW have been involved in revising the manuscript for important intellectual content. All authors read and approved the final manuscript.

## Pre-publication history

The pre-publication history for this paper can be accessed here:

http://www.biomedcentral.com/1471-2296/12/117/prepub

## Supplementary Material

Additional file 1 **Mean scores and factor loadings of the items of the SCOPE questionnaire (43 items; n = 294 respondents)**. Factor loadings > 0.40 are shown. The letter "n" in an item-code means that it concerns an item in negative wording. * Questions that were added when adapting Dutch HSOPS into SCOPE. Click here for file
