# Supplementary material for: Patient safety culture measurement in general practice. Clinimetric properties of 'SCOPE'
Source: BMC Fam Pract. 2011 Nov 1;12:117. doi: 10.1186/1471-2296-12-117 (PMC3228702; doi:10.1186/1471-2296-12-117)
Supplement: Additional file 1 — Mean scores and factor loadings of the items of the SCOPE questionnaire (43 items; n = 294 respondents). Factor loadings > 0.40 are shown. The letter "n" in an item-code means that it concerns an item in negative wording. * Questions that were added when adapting Dutch HSOPS into SCOPE. [file 1471-2296-12-117-S1.DOC]

| Factor | Item | Description | Mean | SD | F1 | F2 | F3 | F4 | F5 | F6 | F7 | F8 | Alpha |
| --- | --- | --- | --- | --- | --- | --- | --- | --- | --- | --- | --- | --- | --- |
| 1 Handover and teamwork | G2n | Problems often occur in the exchange of information across disciplines in our practice | 3.63 | 0.84 | 0.51 |  |  |  |  |  |  |  | 0.76 |
|  | G3n * | The fact that patients are treated by different GPs in our practice is causing problems | 3.77 | 0.85 | 0.59 |  |  |  |  |  |  |  |  |
|  | G4n | Disciplines in our practice do not coordinate well with each other | 3.84 | 0.77 | 0.61 |  |  |  |  |  |  |  |  |
|  | G6 * | There is a good exchange of information between GPs at this practice | 3.93 | 0.72 | 0.64 |  |  |  |  |  |  |  |  |
|  | G7 * | There is a good exchange of information between nurses at this practice | 3.94 | 0.74 | 0.62 |  |  |  |  |  |  |  |  |
|  | G8 | Disciplines work well together to provide the best care for patients | 4.00 | 0.71 | 0.69 |  |  |  |  |  |  |  |  |
|  | G9n | Things “fall between the cracks” when transferring patients between different disciplines in this practice | 3.76 | 0.78 | 0.68 |  |  |  |  |  |  |  |  |
|  | G10n * | Important patient care information is often lost because patients see different GPs | 3.89 | 0.89 | 0.56 |  |  |  |  |  |  |  |  |
| 2 Support and fellowship | A1 | People support one another in this practice | 4.12 | 0.65 |  | 0.79 |  |  |  |  |  |  | 0.72 |
|  | A3 | When a lot of work needs to be done quickly, we work together as a team to get the work done | 4.02 | 0.70 |  | 0.75 |  |  |  |  |  |  |  |
|  | A4 | In this practice, people treat each other with respect. | 4.20 | 0.64 |  | 0.73 |  |  |  |  |  |  |  |
|  | A9 | Mistakes have led to positive changes here | 3.58 | 0.69 |  | 0.53 |  |  |  |  |  |  |  |
|  | A11 | When one area in this practice gets really busy, others help out | 3.74 | 0.76 |  | 0.64 |  |  |  |  |  |  |  |
| 3 Communication openness | A8n | Staff feel like their mistakes are held against them | 4.10 | 0.94 |  |  | 0.51 |  |  |  |  |  | 0.66 |
|  | A12n | When an event is reported, it feels like the person is being written up, not the problem | 3.60 | 0.81 |  |  | 0.51 |  |  |  |  |  |  |
|  | A15n | Staff worry that mistakes they make are kept in their personnel file | 4.07 | 0.80 |  |  | 0.67 |  |  |  |  |  |  |
|  | C2 | Staff will freely speak up if they see something that may negatively affect patient care | 4.33 | 0.69 |  |  | 0.71 |  |  |  |  |  |  |
|  | C4 | Staff feel free to question the decisions or actions of those with more authority | 3.78 | 0.83 |  |  | 0.66 |  |  |  |  |  |  |
|  | C6n | Staff are afraid to ask questions when something does not seem right | 3.97 | 0.88 |  |  | 0.58 |  |  |  |  |  |  |
| 4 Feedback about and learning from error | C1 | We are given feedback about changes put into place based on event reports | 4.08 | 0.91 |  |  |  | 0.64 |  |  |  |  | 0.82 |
|  | C3 | We are informed about errors that happen in this practice | 4.01 | 0.85 |  |  |  | 0.72 |  |  |  |  |  |
|  | C5 | In this practice, we discuss ways to prevent errors from happening again | 4.18 | 0.72 |  |  |  | 0.77 |  |  |  |  |  |
|  | C7 * | GPs discuss errors that happened in this practice with each other | 4.15 | 0.80 |  |  |  | 0.74 |  |  |  |  |  |
|  | C8 * | GPs discuss errors that happened in this practice with other staff | 3.82 | 0.87 |  |  |  | 0.74 |  |  |  |  |  |
|  | C9 * | We are given personal feedback about our own event reports | 3.94 | 0.90 |  |  |  | 0.72 |  |  |  |  |  |
| 5 Intention to report events | D1 | When a mistake is made, but is caught and corrected before affecting the patient, how often is this reported? | 3.69 | 1.09 |  |  |  |  | 0.85 |  |  |  | 0.85 |
|  | D2 | When a mistake is made, but has no potential to harm the patient, how often is this reported? | 3.77 | 1.01 |  |  |  |  | 0.91 |  |  |  |  |
|  | D3 | When a mistake is made that could harm the patient, but does not, how often is this reported? | 4.14 | 0.92 |  |  |  |  | 0.86 |  |  |  |  |
| 6 Adequate procedures and adequate staffing | A2 | We have enough staff to handle the workload | 3.65 | 0.96 |  |  |  |  |  | 0.56 |  |  | 0.66 |
|  | A10n | Staff in this practice work longer hours than is best for patient care | 3.95 | 0.79 |  |  |  |  |  | 0.59 |  |  |  |
|  | A14n | We work in “crisis mode” trying to do too much, too quickly | 3.73 | 0.88 |  |  |  |  |  | 0.74 |  |  |  |
|  | A16n | We have patient safety problems in this practice | 3.97 | 0.77 |  |  |  |  |  | 0.57 |  |  |  |
|  | A5n | It is just by chance that more serious mistakes don’t happen around here | 3.74 | 0.98 |  |  |  |  |  | 0.55 |  |  |  |
|  | A7n | We use more agency/temporary staff than is best for patient care | 4.34 | 0.81 |  |  |  |  |  | 0.57 |  |  |  |
|  | A17 | Our procedures and systems are good at preventing errors from happening | 3.43 | 0.78 |  |  |  |  |  | 0.41 |  |  |  |
| 7 Overall perceptions of patient safety management | A6 | We are actively doing things to improve patient safety | 3.47 | 0.84 |  |  |  |  |  |  | 0.58 |  | 0.67 |
|  | B5 | My supervisor/manager provides a work climate that promotes patient safety | 3.80 | 0.59 |  |  |  |  |  |  | 0.77 |  |  |
|  | B6 | The actions of my supervisor/manager show that patients safety is top priority | 3.51 | 0.69 |  |  |  |  |  |  | 0.80 |  |  |
|  | B7n | My supervisor/manager seems interested in patient safety only after an adverse event happens | 3.88 | 0.76 |  |  |  |  |  |  | 0.68 |  |  |
| 8 Expectations and actions of managers | B1 | My supervisor/manager says a good word when he/she sees a job done according to established patient safety procedures | 3.63 | 0.74 |  |  |  |  |  |  |  | 0.71 | 0.72 |
|  | B2 | My supervisor/manager seriously considers staff suggestions for improving patient safety | 3.96 | 0.51 |  |  |  |  |  |  |  | 0.74 |  |
|  | B3n | Whenever pressure builds up, my supervisor/manager wants us to work faster, even if it means taking shortcuts | 3.89 | 0.76 |  |  |  |  |  |  |  | 0.72 |  |
|  | B4n | My supervisor/manager overlooks patient safety problems that happen over and over | 3.76 | 0.83 |  |  |  |  |  |  |  | 0.77 |  |
| Item deleted | A13 | After we make changes to improve patient safety, we evaluate their effectiveness |  |  |  |  |  |  |  |  |  |  |  |
| Item deleted | G1 | Important patient care information is often lost during shift changes from day-care to out-of-hours care |  |  |  |  |  |  |  |  |  |  |  |
| Item deleted | G5 | Important patient care information is often lost during shift changes from out-of-hours care to day-care |  |  |  |  |  |  |  |  |  |  |  |

**Additional File 1**

Title:

Mean scores and factor loadings of the items of the SCOPE questionnaire (43 items; n= 294 respondents)

*Legends:*

*Factor loadings >0.40 are shown. The letter “n” in an item-code means that it concerns an item in negative wording.*

** Questions that were added when adapting Dutch HSOPS into SCOPE.*
